# The Impact of an Electronic Portal on Patient Encounters in Primary Care: Interrupted Time-Series Analysis

**DOI:** 10.2196/43567

**Published:** 2023-02-06

**Authors:** Karen Ferguson, Mark Fraser, Meltem Tuna, Charles Bruntz, Simone Dahrouge

**Affiliations:** 1 Department of Family Medicine University of Ottawa Ottawa, ON Canada; 2 West Carleton Family Health Team Carp, ON Canada; 3 ICES Ottawa, ON Canada; 4 Ottawa Hospital Research Institute Ottawa, ON Canada; 5 Champlain Family Health Teams Ottawa, ON Canada; 6 Bruyère Research Institute Ottawa, ON Canada

**Keywords:** electronic health records, health care utilization, patient portals, primary care, medical informatics, office visits, electronic, patient, online applications, virtual care, messaging, clinical, age, sex, education

## Abstract

**Background:**

Electronic patient portals are online applications that allow patients access to their own health information, a form of asynchronous virtual care. The long-term impact of portals on the use of traditional primary care services is unclear, but it is an important question at this juncture, when portals are being incorporated into many primary care practices.

**Objective:**

We sought to investigate how an electronic patient portal affected the use of traditional, synchronous primary care services over a much longer time period than any existing studies and to assess the impact of portal messaging on clinicians’ workload.

**Methods:**

We conducted a propensity-score–matched, open-cohort, interrupted time-series evaluation of a primary care portal from its implementation in 2010. We extracted information from the electronic medical record regarding age, sex, education, income, family health team enrollment, diagnoses at index date, and number of medications prescribed in the previous year. We also extracted the annual number of encounters for up to 8 years before and after the index date and provider time spent on secure messaging through the portal.

**Results:**

A total of 7247 eligible portal patients and 7647 eligible potential controls were identified, with 3696 patients matched one to one. We found that portal registration was associated with an increase in the number of certain traditional encounters over the time period surrounding portal registration. Following the index year, there was a significant jump in annual number of visits to physicians in the portal arm (0.42 more visits/year vs control, *P*<.001) but not for visits to nurse practitioners and physician assistants. The annual number of calls to the practice triage nurses also showed a greater increase in the portal arm compared to the control arm after the index year (an additional 0.10 calls, *P*=.006). The average provider time spent on portal-related work was 5.7 minutes per patient per year.

**Conclusions:**

We found that portal registration was associated with a subsequent increase in the number of some traditional encounters and an increase in clerical workload for providers. Portals have enormous potential to truly engage patients as partners in their own health care, but their impact on use of traditional health care services and clerical burden must also be considered when they are incorporated into primary care.

## Introduction

Electronic patient portals are online applications that allow patients access to their own health information, a form of asynchronous virtual care. There has been a great deal of recent interest in patient portals, accompanied by increasing technology adoption by both clinicians and patients [1-3]. The COVID-19 pandemic has also highlighted the importance of virtual care, an area already identified as a national health care priority [4]. Although portal features vary, the safe communication channels in portals may provide alternative ways for patients to obtain services traditionally provided in person, such as renewing prescriptions, sending and receiving secure messages, obtaining test results, and booking appointments [5]. A recent survey indicated that approximately 20% of Canadians had accessed some of their own medical information electronically, and that almost 80% were interested in doing so [6]. However, that survey did not specifically address portals or patient access to their medical information in primary care practice settings, and we are not aware of any studies examining Canadian portal adoption in primary care. Our understanding of the potential value of patient portals is nascent, with portals expected to contribute to more authentic collaboration between clinicians and patients.

The long-term impact of portals on traditional primary care services is unclear, but it is an important question at this juncture, when portals are being incorporated into primary care practices. Many studies reporting on the impact of portals on the use of traditional services evaluated systems that only provided options for web messaging or booking appointments [7-13]. All existing studies that investigated portals with more diverse features were conducted in medical networks, such as health maintenance organizations, where the portals provided access across sectors, including primary care, specialty care, and hospital care; these studies may not be relevant to portals incorporated into exclusively primary care practices. Past studies also reported inconsistent findings regarding the impact of portals on traditional health care use. Some studies demonstrated an increase in visits [14-16] or telephone calls [17]. Others demonstrated no change in visits [18], a reduction in visits [19], or a reduction in hospital readmissions [20]. All these studies also had limited time frames, examining only the period 12 to 30 months after portal registration.

To our knowledge, no long-term evaluation of the impact of a primary care patient portal on traditional health care use has been conducted to date. Providers have expressed interest in patient portals but also concerns regarding medicolegal risk and clerical workload [21]. Some have described an increased clerical burden associated with portals as part of the electronic health environment [12,22]. For instance, a qualitative study examining online patient access to their own health records found that providers felt that their workload had increased as a result [23], while another found that some providers anticipated fewer administrative requests for information when patients had access to their own health records [24]. One study found that online patient access to encounter notes did not significantly affect physician workload [25], although others have described high volumes of portal messages sent by patients [26]. However, no studies have actually tracked the provider time spent specifically on portal-related work. There have also not been any large studies of the impact of electronic patient portals in a Canadian setting. We sought to investigate how an electronic patient portal affects traditional, synchronous, primary care health care use over a much longer time period than any existing studies, and to assess the impact of portal messaging on clinicians’ workload.

## Methods

We conducted a propensity-score–matched, open-cohort, interrupted time-series (ITS) evaluation of a primary care portal from its implementation in 2010.

### Setting and Study Participants

The practice was a semirural interprofessional clinic in southeastern Ontario, Canada, where 12 family physicians and other allied health providers provide comprehensive primary care under a single-payer model. Under this publicly funded model, physician compensation is primarily through capitation payments for rostered patients. The primary care patient portal initially offered access to laboratory results, the ability to enter vital signs such as blood pressure measurements, and the ability to view when certain screening maneuvers were due. Additional features were introduced over time, including the ability to receive secure messages (in 2012), send secure messages (in 2015), book appointments (in 2016), and renew prescriptions (in 2018.) All practice patients were invited to join the portal via email, posters, and telephone reminders and at in-person encounters.

We collected data for all practice patients except those seen exclusively for focused care (eg, obstetrical care). We retained data from all patients only for the period they were aged 18 years or older. Among patients who adopted the portal, we excluded those for whom we did not have at least one year of data prior to and following their portal registration (ie, index) date. For non–portal patients, we excluded those who did not have at least two consecutive years of data between 2009 and 2019.

### Matching

We calculated propensity scores to estimate the probability of individuals registering for the portal using logistic regression [27,28]. Propensity scores were derived based on sex, age, whether the patient was rostered to the family health team, the presence of specific diagnoses on the index date, and the number of in-person and telephone encounters, as well as the number of unique medications prescribed in the 12 months prior to the index date. During the study time period, all appointments with medical doctors (MDs), nurse practitioners (NPs), and physician assistants (PAs) were in person. Since education and income level were recorded for approximately a third of patients, these measures were not included in the propensity score matching. Control patients were entered into the equation for each year they were eligible (ie, for each year they had at least one year of data prior to and after the index date), with their corresponding profile for that year. July 1 of that year was considered the index date.

### Variables, Data Sources, and Measurement

The study period was January 2002 through December 2019. We extracted electronic medical record information on patient age, sex, education, income, enrollment with the practice, and presence or absence of specific diagnoses on the index date. We also extracted the dates of in-person encounters with MDs, NPs, and PAs; dates of triage calls (TCs) to the practice triage nurses; and prescription dates and prescribed medications. Prescribed medications included only those that were identified as distinct medications using Anatomical Therapeutic Chemical codes. Diagnoses were defined based on diagnostic codes, using the earliest date when the diagnostic code was applied.

In order to study the clinician workload associated with the portal, two providers (KF and MF) time stamped their portal messages between February 20, 2020, and February 25, 2021. This allowed us to estimate the average provider time spent per message. We also collected the total number of portal messages sent by all providers to all portal patients between January 1, 2019, and December 31, 2019, in order to determine the average amount of time spent per patient on portal-related work.

### Analyses

We described the profile of eligible patients prior to matching on their index date for portal patients on July 1 of the median year for which they were eligible to be matched for non–portal patients and again for the matched patients on their index date in both arms. The main study outcome was the frequency of in-person encounters with MDs, NPs, or PAs, as well as frequency of TCs. We used an ITS design to evaluate the impact of portal registration on use of these traditional health care services over time and compared use by portal users to their matched controls. We present the results in the usual ITS format, defining time relative to the index date with year 0 representing the 12 months preceding and including the index date and each time unit representing a 12-month interval. The ITS model includes time as a linear variable to model for an underlying linear time trend and the portal enrollment (ie, the intervention) as a dummy variable. Intervention and time interaction is also included in the model to identify the effect of the intervention on both arms (ie, portal and non–portal) over time.

We plotted the annual number of in-person encounters with MDs, in-person encounters with NPs or PAs, and TCs across time and overlaid the estimates derived from the ITS equations. During the study time period, all appointments with MDs, NPs, and PAs were in person. Because the year-0 results showed a spike in service use in both study groups, likely related to the attribution of the index date, we excluded that year from the ITS model. Also, although the spike in service use at year 1 in the portal arm may represent a transient change in behavior associated with the initial adoption, we also excluded this from the ITS to obtain a more reliable estimate of the impact of portal adoption over time, recognizing that this approach omits significant use; this should be considered in result interpretation.

We also depicted the number of visits per calendar year for patients who adopted the portal grouped by year of portal registration to demonstrate the pattern of changes in these visits over time for the intervention arm.

### Ethics Approval

Ethics approval was received from the Bruyère Research Ethics Board (M16-20-012).

## Results

### Matching

**Figure 1:**
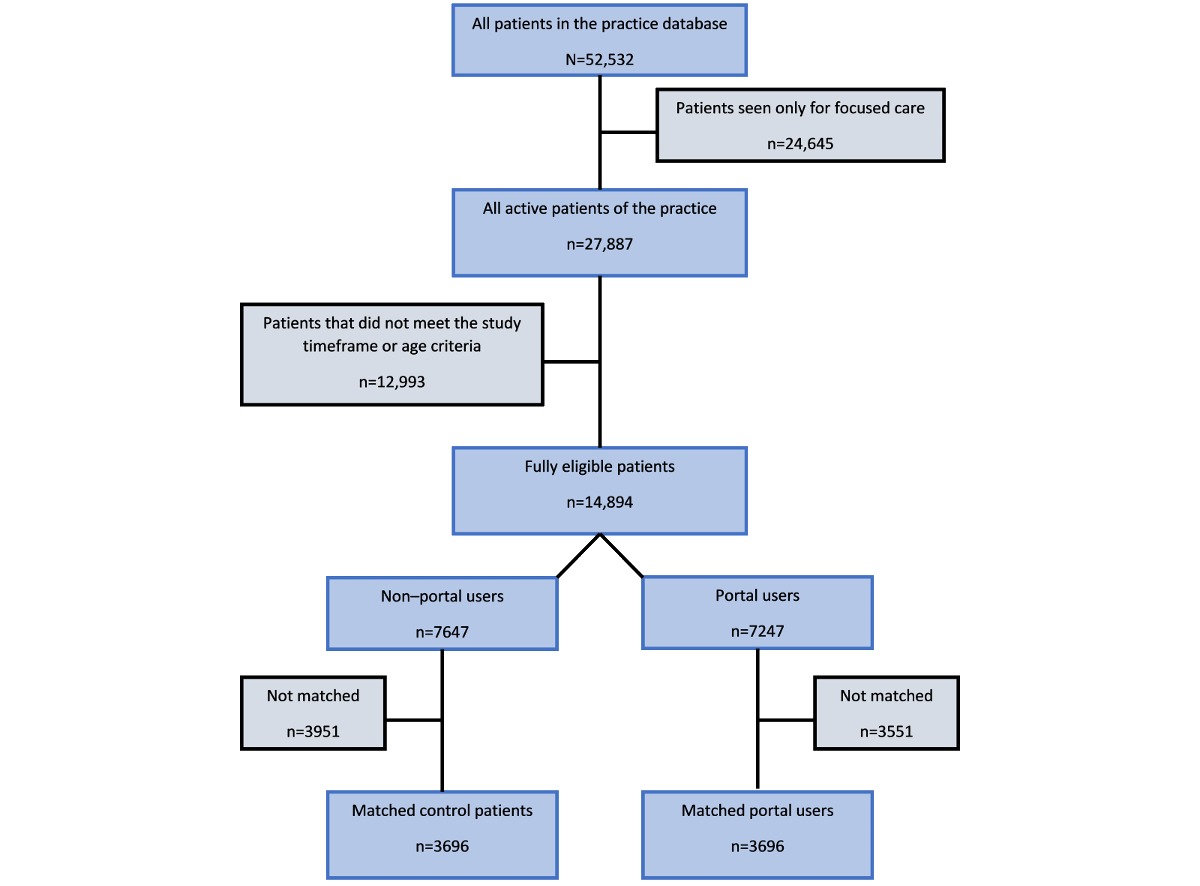
Study patient selection.

Of the 14,894 patients who met the study criteria, 7247 (48.7%) were portal participants. Of these, 3696 were matched one to one with a control patient (Figure 1). The profile of all eligible patients before and after propensity matching is shown in Table 1. Before matching, portal users differed from non–portal users, but after matching, the mean propensity scores of the 2 groups and their index years, the prevalence of chronic conditions, sex, rostering status, and total visits and medications in the previous years showed good agreement. Income and education levels, which could not be included in the propensity score derivation because of poor data completeness, remained higher in the portal group.

We used a caliper of 0.2 for matching and limited the potential matching pool for each portal patient to non–portal patients with an index date that was within 1 year of the portal patient’s index date. We identified all potential controls for each portal patient and assigned matches prioritizing first portal patients who had a unique match, then non–portal patients who had a unique portal match. We repeated this after each match to minimize loss of controls. When more than one match was possible, we attributed the control patient whose propensity score was closest to the portal patient’s score. The balance of baseline covariates between the matched portal users and non–portal users was assessed using standardized differences, with values <0.1 representing negligible differences.

**Table 1:** Portal and control patients before and after matching.

Variable			Portal, index date (n=7247)	Non–portal, median index date (n=7647)	Total (n=14,894)	*P* value		Standard difference		Portal, index date (n=3696)			Control, index date (n=3696)		Total (n=7392)		*P* value		Standard difference		
**On index date**																					
	Propensity score, mean (SD)		0.21 (0.10)	0.10 (0.08)	0.15 (0.11)	<.001		1.28		0.20 (0.10)			0.20 (0.10)		0.20 (0.10)		.82		0.01		
	**Propensity score groups (participants), n (%)**						<.001											.10			
		0	291 (4)	2687 (35.1)	2978 (20)			0.85		738 (20)			740 (20)		1478 (20)				0		
		1	947 (13.1)	2032 (26.6)	2979 (20)			0.34		735 (19.9)			743 (20.1)		1478 (20)				0.01		
		2	1349 (18.6)	1631 (21.3)	2980 (20)			0.07		743 (20.1)			736 (19.9)		1479 (20)				0		
		3	2010 (27.7)	968 (12.7)	2978 (20)			0.38		740 (20)			742 (20.1)		1482 (20)				0		
		4	2650 (36.6)	329 (4.3)	2979 (20)			0.87		740 (20)			735 (19.9)		1475 (20)				0		
	**Index year^a^ (participants), n (%)**						<.001											.99			
		2010	1-5^b^	148-152^b^	153 (1)			0.2		1-5^b^			1-5^b^		1-5^b^				0		
		2011	1335 (18.4)	522 (6.8)	1857 (12.5)			0.35		654 (17.7)			654 (17.7)		1308 (17.7)				0		
		2012	1240 (17.1)	390 (5.1)	1630 (10.9)			0.39		579 (15.7)			596 (16.1)		1175 (15.9)				0.01		
		2013	948 (13.1)	349 (4.6)	1297 (8.7)			0.3		461 (12.5)			452 (12.2)		913 (12.4)				0.01		
		2014	531 (7.3)	3717 (48.6)	4248 (28.5)			1.04		296-300^b^			275-279^b^		576-580^b^				0.02		
		2015	664 (9.2)	838 (11)	1502 (10.1)			0.06		344 (9.3)			353 (9.6)		697 (9.4)				0.01		
		2016	970 (13.4)	670 (8.8)	1640 (11)			0.15		515 (13.9)			518 (14)		1033 (14)				0		
		2017	793 (10.9)	681 (8.9)	1474 (9.9)			0.07		464 (12.6)			455 (12.3)		919 (12.4)				0.01		
		2018	761-765^b^	328-332^b^	1093 (7.3)			0.24		378 (10.2)			388 (10.5)		766 (10.4)				0.01		
	Age at index date (years), mean (SD)		48.9 (14.9)	45.2 (19.3)	47.0 (17.4)	<.001		0.22		46.56 (15.17)			46.18 (15.93)		46.37 (15.56)		.29		0.02		
	**Sex (participants), n (%)**						<.001		0.22									.74		0.01	
		Female	4334 (59.8)	3748 (49)	8082 (54.3)					2104 (56.9)			2090 (56.5)		4194 (56.7)						
		Male	2913 (40.2)	3899 (50.1)	6812 (45.7)					1592 (43.1)			1606 (43.5)		3198 (43.3)						
	Rostered, n (%)		7162 (98.8)	7224 (94.5)	14,386 (96.6)	<.001		0.24		3665 (99.2)			3652 (98.8)		7317 (99)		.13		00.04		
	Coronary artery disease, n (%)		243 (3.4)	268 (3.5)	511 (3.4)	.61		0.01		68 (1.8)			79 (2.1)		147 (2)		.36		0.02		
	Congestive heart failure, n (%)		79 (1.1)	112 (1.5)	191 (1.3)	.04		0.03		24 (0.6)			31 (0.8)		55 (0.7)		.34		0.02		
	Chronic obstructive pulmonary disease, n (%)		128 (1.8)	235 (3.1)	363 (2.4)	<.001		0.09		43 (1.2)			54 (1.5)		97 (1.3)		.26		0.03		
	Diabetes mellitus, n (%)		440 (6.1)	482 (6.3)	922 (6.2)	.56		0.01		149 (4)			154 (4.2)		303 (4.1)		.77		0.01		
	Hypertension, n (%)		1343 (18.5)	1190 (15.6)	2533 (17)	<.001		0.08		495 (13.4)			474 (12.8)		969 (13.1)		.47		0.02		
	**Income level (CAD $)^c,d^ (participants), n (%)**						<.001											<.001			
		<40,000	233 (8.7)	432 (15.8)	665 (12.3)			0.22		121 (8.7)			150 (12.2)		271 (10.4)				0.11		
		40,000-60,000	357 (13.3)	484 (17.7)	841 (15.5)			0.12		173 (12.5)			218 (17.7)		391 (14.9)				0.15		
		60,000-100,000	846 (31.6)	836 (30.5)	1682 (31)			0.02		437 (31.5)			397 (32.3)		834 (31.9)				0.02		
		>100,000	1242 (46.4)	989 (36.1)	2231 (41.2)			0.21		657 (47.3)			465 (37.8)		1122 (42.9)				0.19		
	**Education level^c^ (participants), n (%)**						<.001											<.001			
		High school or less	526 (21)	1037 (39.7)	1563 (30.5)			0.41		284 (21.7)			380 (32)		664 (26.6)				0.23		
		College	812 (32.4)	745 (28.5)	1557 (30.4)			0.08		397 (30.4)			381 (32.1)		778 (31.2)				0.04		
		University or more	1363 (50.5)	979 (35.5)	2342 (42.9)			0.31		626 (47.9)			427 (35.9)		1053 (42.2)				0.24		
**In the 12 months prior to index date**																					
	**Medical doctor visits^e^, n (%)**						<.001											.07			
		0	1068 (14.7)	3328 (43.5)	4396 (29.5)			0.67		732 (19.8)			648 (17.5)		1380 (18.7)				0.06		
		1-2	3758 (51.9)	2548 (33.3)	6306 (42.3)			0.38		1969 (53.3)			2079 (56.3)		4048 (54.8)				0.06		
		3-5	1809 (25)	1251 (16.4)	3060 (20.5)			0.21		776 (21)			750 (20.3)		1526 (20.6)				0.02		
		6-10	532 (7.3)	437 (5.7)	969 (6.5)			0.07		189 (5.1)			187 (5.1)		376 (5.1)				0		
		>11	80 (1.1)	83 (1.1)	163 (1.1)			0		30 (0.8)			32 (0.9)		62 (0.8)				0.01		
	**Nurse practitioner or physician assistant visits^e^, n (%)**						<.001											.34			
		0	3959 (54.6)	5213 (68.2)	9172 (61.6)			0.28		2272 (61.5)			2297 (62.1)		4569 (61.8)				0.01		
		1-2	2655 (36.6)	2009 (26.3)	4664 (31.3)			0.22		1232 (33.3)			1234 (33.4)		2466 (33.4)				0		
		>3	633 (8.7)	425 (5.6)	1058 (7.1)			0.12		192 (5.2)			165 (4.5)		357 (4.8)				0.03		
	**Calls to triage nurses^e^, n (%)**						<.001											.40			
		0	4990 (68.9)	5886 (77)	10,876 (73)			0.18		2827 (76.5)			2871 (77.7)		5698 (77.1)				0.03		
		1-2	1898 (26.2)	1465 (19.2)	3363 (22.6)			0.17		759 (20.5)			728 (19.7)		1487 (20.1)				0.02		
		>3	359 (5)	296 (3.9)	655 (4.4)			0.05		110 (3)			97 (2.6)		207 (2.8)				0.02		
	**Medications prescribed^e^, n (%)**						<.001											.78			
		0	1705 (23.5)	3429 (44.8)	5134 (34.5)			0.46		1131 (30.6)			1132 (30.6)		2263 (30.6)				0		
		1-2	2522 (34.8)	2042 (26.7)	4564 (30.6)			0.18		1399 (37.9)			1439 (38.9)		2838 (38.4)				0.02		
		3-5	1928 (26.6)	1272 (16.6)	3200 (21.5)			0.24		817 (22.1)			792 (21.4)		1609 (21.8)				0.02		
		6-10	906 (12.5)	701 (9.2)	1607 (10.8)			0.11		288 (7.8)			281 (7.6)		569 (7.7)				0.01		
		>11	186 (2.6)	203 (2.7)	389 (2.6)			0.01		61 (1.7)			52 (1.4)		113 (1.5)		.82		0.02		

^a^Index date for unmatched control patients: July 1 of the median year of their eligible time period.

^b^The value of n for 2010 was smaller than 6. The cells for 2010, 2014, and 2018 therefore do not have precise values due to ethics agreements.

^c^Income and education level were not available for all patients.

^d^CAD $1.00=US $0.75 on January 12, 2023.

^e^Number of medications prescribed and number of encounters refer to the 12-month period prior to the index date. The medication records were mapped to the Drug Product Database to assign Anatomical Therapeutic Chemical codes and schedules based on drug identification numbers. We excluded 12.5% of medication records, including records for medications classified as “over-the-counter” or “ethical” in the schedules from the Drug Product Database (4.9%) and medications reclassified manually as “over-the-counter,” “other,” or “N/A” (4.7%). Medications with no drug identification number were classified manually. Those which could not be attributed a drug identification number were also excluded (2.9%). Variable categorization for number of visits, telephone calls and medications was based on clinical judgement and the number of participants in each category.

### Analyses

**Figure 2:**
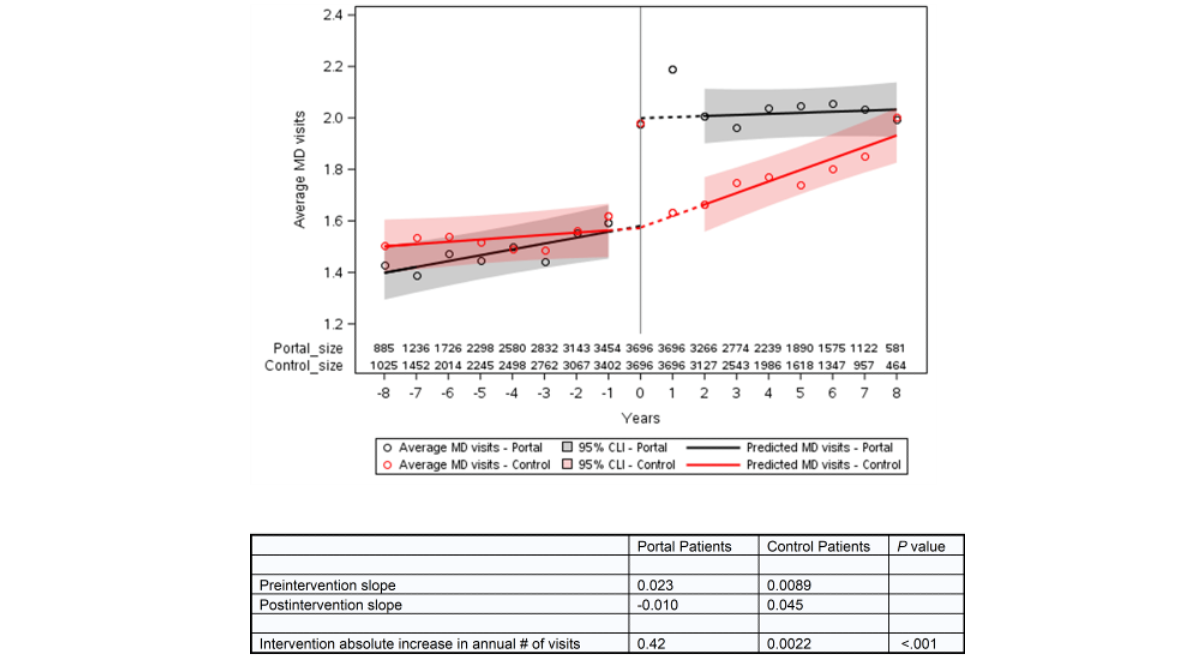
Interrupted time series for MD face-to-face visits for portal patients versus controls. The intercepts (*P*=.86) and slopes (*P*=.15) prior to the index year were similar in the control and portal arms. After the index year, there was no significant change in the number of MD visits in the control arm. However, in the portal arm, there was a significant jump in number of visits and a new intercept (0.42 more visits/year vs control, *P*<.001). The slope for MD visits increased after the index date in the control arm but became negative in the portal arm, representing an annual reduction of 0.054 visits per year for the portal arm compared to the control arm (*P*=.001). The two slopes would be expected to cross after 10 years. CLI: confidence limit interval; MD: medical doctor.

**Figure 3:**
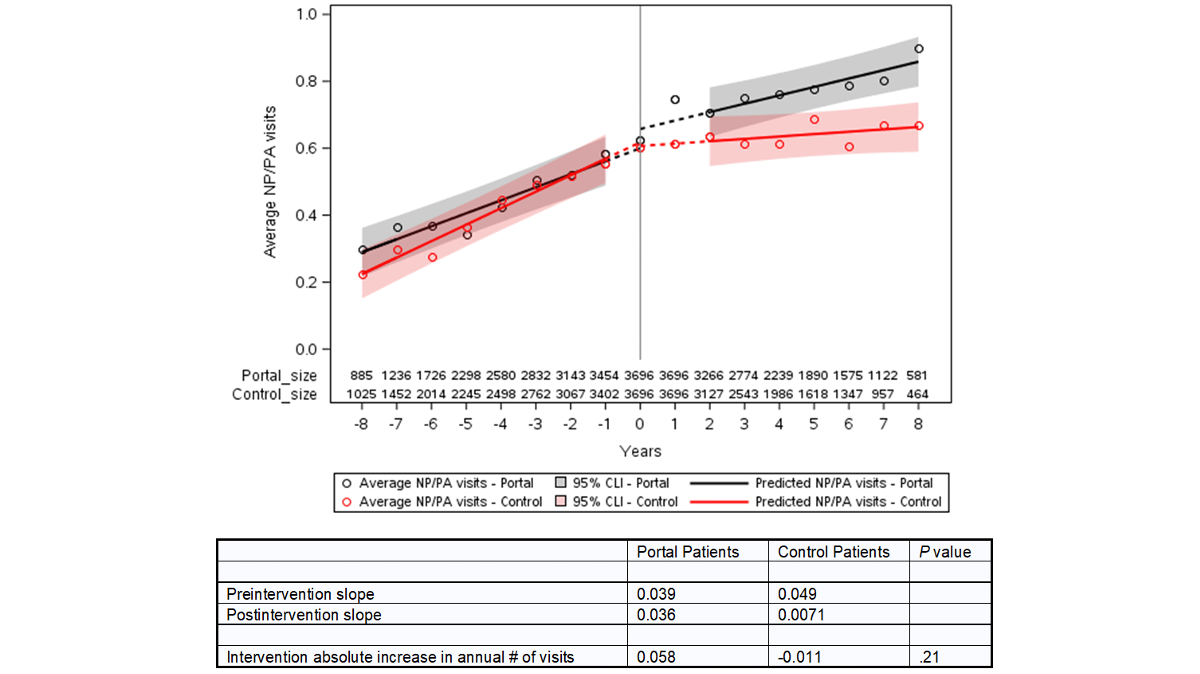
Interrupted time series for nurse practitioner or physician assistant face-to-face visits for portal patients versus controls. The intercepts (*P*=.59) and slopes (*P*=.12) prior to the index year were similar in the control and portal arms. After the index year, there was not a significant change in the number of nurse practitioner or physician assistant visits in the portal arm compared to the control arm (*P*=.21). The slope flattened after the index date in the control arm, but it was relatively unchanged in the portal arm, demonstrating an annual increase of 0.028 visits per year in the portal arm compared to the control arm (*P*=.01). CLI: confidence limit interval; PA: physician assistant; NP; nurse practitioner.

**Figure 4:**
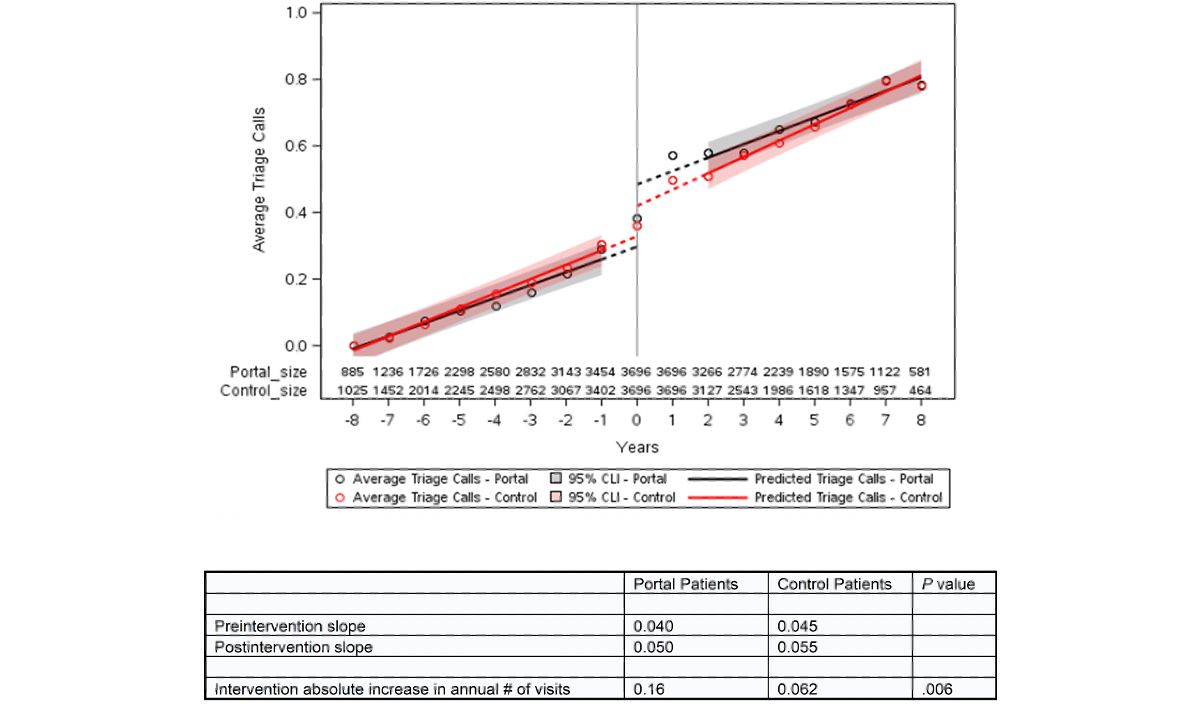
Interrupted time series for triage calls for portal patients versus controls. The intercepts (*P*=.10) and slopes (*P*=.26) prior to the index year were similar in the control and portal arms. The number of triage calls following the index year showed a higher value than anticipated based on the preindex slope in the control arm (0.062 more calls annually, *P*=.02), but a significantly greater jump after the index year in the portal arm (0.10 more calls annually, *P*=.006). The slopes for annual triage calls were similar in the pre- and postindex periods for both the control arm and portal arm. CLI: confidence limit interval.

We plotted the number of visits in relation to the index year for portal and control patients, the estimated slopes for the years before and after the transition, and the shift in visits at the index date derived from the ITS equation (Figures 2-4). The information for the years prior to the index date for portal patients demonstrates their annual visits for the years prior to their registration on the portal. The index date for the control patients was assigned to be within 1 year of the portal’s patient index date in order to control for temporal factors such as health care use trends. The outputs of the ITS analyses are provided in Table 2. The intercepts and slopes prior to the index year were similar in the control and portal arms for MDs, NPs/PAs, and TCs (*P*>.05). After the index year, there was a significant jump in MD visits in the portal arm (0.42 more visits/year vs control, *P*<.001) but not for NP or PA visits. The TCs also showed a greater increase in visits in the portal arm compared to the control arm after the index year (0.102 more visits/year vs control, *P*=.006).

**Table 2:** Outputs of the interrupted time series. “Annual visits” indicates slope; “period” indicates the pre- or postindex period.

Variable	Medical doctor visits			Nurse practitioner or physician assistant visits			Triage calls	
	Estimate	*P* value	Estimate		*P* value	Estimate		*P* value
Intercept^a^	1.572	<.001	0.618		<.001	0.335		<.001
Annual visits (slope)^b^	0.009	.19	0.049		<.001	0.045		<.001
Period (before or after index)^c^	0.002	.97	-0.011		.77	0.062		.02
Annual visits × period^d^	0.036	.002	-0.042		<.001	0.010		.05
Portal^e^	0.008	.86	-0.018		.59	–0.033		.10
Portal × annual visits^f^	0.014	.15	-0.010		.12	–0.005		.26
Portal × period^g^	0.417	<.001	0.069		.21	0.102		.006
Portal × annual visits × period^h^	–0.054	.001	0.028		.01	–0.005		.46

^a^Control arm intercept.

^b^Pre–index date slope of annual visits for the control arm.

^c^Change in number of visits in year 2 post–index date relative to that anticipated from preindex slope for the control arm.

^d^Change in the slope of annual visits in the postindex period relative to the preindex period for the control arm.

^e^Difference between the portal arm and control arm in the intercept.

^f^Difference between the portal arm and control arm in the pre–index date slope of annual visits.

^g^Difference between the portal arm and control arm in the change in number of visits in year 2 post–index date relative to that anticipated from preindex slope.

^h^Difference between the portal arm and control arm in the change of the slope of annual visits in the postindex period relative to the preindex period.

**Figure 5:**
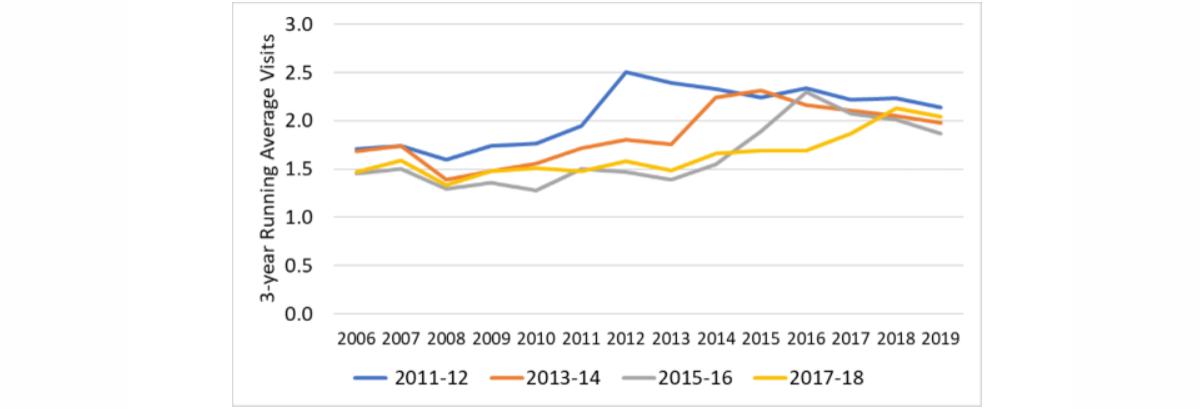
Number of visits to medical doctors per calendar year for each patient group (registered on the portal in 2011-2012, 2013-2014, 2015-2016, and 2017-2018). To reduce noise, the number of visits represents the running average of that year, the previous year, and the following year. The MD visits showed a slight increase in the number of visits in the years immediately following portal registration, followed by an apparent drop in annual rate of visits.

**Figure 6:**
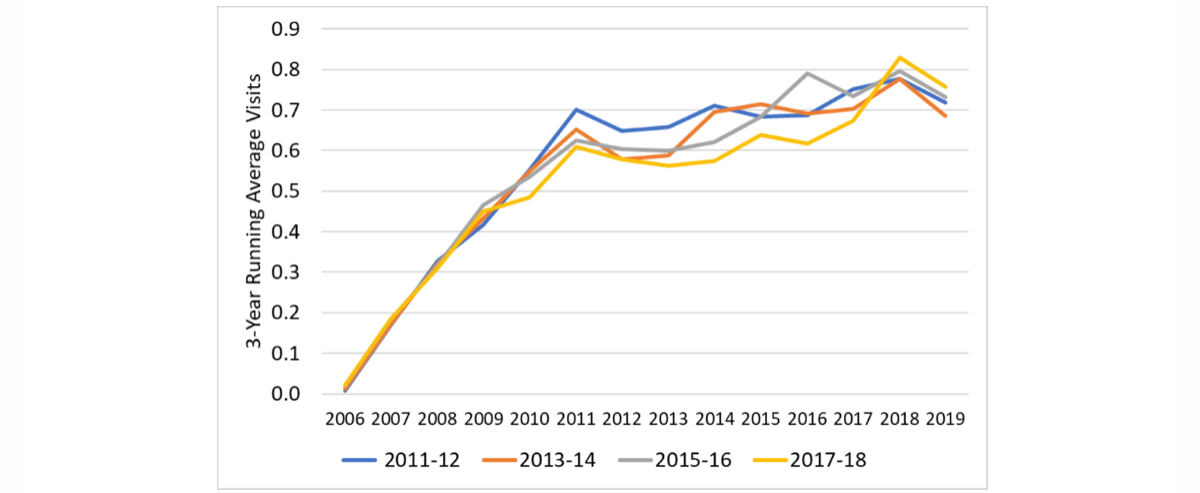
Number of visits to nurse practitioners or physician assistants for each patient group (registered on the portal in 2011-2012, 2013-2014, 2015-2016, and 2017-2018). To reduce noise, the number of visits represents the running average of that year, the previous year, and the following year. The NP and PA visits began in 2006 and show a rapid rise in the number of visits until 2010, then a considerable flattening of that slope afterwards with a potential small spike following the year of registration.

**Figure 7:**
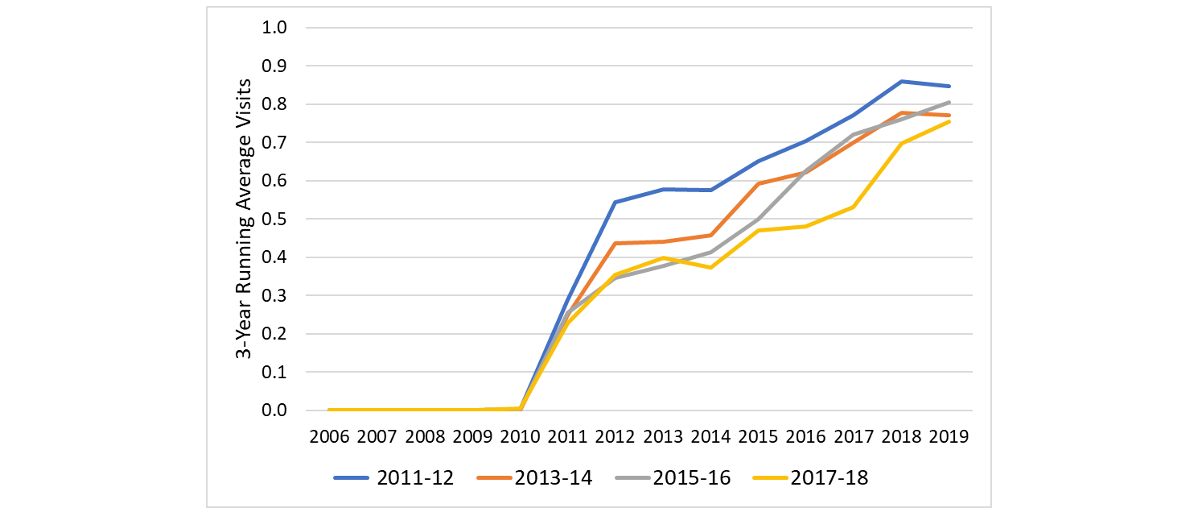
Number of triage calls per calendar year for each patient group (registered on the portal in 2011-2012, 2013-2014, 2015-2016, and 2017-2018). To reduce noise, the number of visits represents the running average of that year, the previous year, and the following year. The nurse triage calls were introduced in 2010 and show a consistent rise in frequency over time with a small increase in calls associated with the year of portal registration.

We also plotted the visit rates for each year for patients having enrolled in the portal, grouped by year of portal registration (Figures 5-7).

The 2 physicians who time stamped 2061 portal messages spent an average of 3.83 minutes on each message. We also extracted the total number of portal messages sent by all providers between January 1 and December 31, 2019, and found that an average of 1.49 messages were sent to each portal patient in the practice. Thus, the average amount of provider time devoted to portal messages was estimated to be 5.7 minutes per portal patient per year.

## Discussion

### Main Findings

Our findings suggest that portal registration is associated with an increase in service use, but that some reductions may be expected over subsequent years. Compared to matched controls, portal registration was associated with a significant initial increase in the number of in-person MD encounters and telephone calls, but a subsequent drop in the rate of MD visits and increase in NP visits over time. MDs spent an estimated 5.7 minutes per patient annually to respond to portal messages.

### Limitations and Comparison With Prior Work

We believe that ours is the first study to examine the trend in encounters after portal registration over an extended time span and the first study to examine the impact of an exclusively primary care portal on traditional health care usage. It is possible that the observed increase in encounters was due to differences between the two groups that were not captured in the propensity matching. For instance, patients might have registered on the portal when they developed a new health concern, anticipating an increased requirement for health services. The reason for the gradual decrease in MD visits but increase in NP visits that took place after the initial jump in MD visits associated with portal registration is difficult to determine without further study. It is possible that patients initially presented to their own physician after sending them a portal message or viewing results, but the physician shared follow-up care with the nurse practitioner or physician assistant.

There may have been differences in areas such as electronic literacy or internet access that were not identified. It is also possible that the higher frequency of in-person encounters after portal registration was due to increased engagement by patients in their health. For instance, access to laboratory results may have generated questions from patients [29]. Increased awareness of being due for cancer screening or diabetes or blood pressure monitoring may have resulted in a higher number of encounters but improved quality of care or patient satisfaction. We did not examine these areas as they were beyond the scope of this study, but they would benefit from future research. While some past studies demonstrated improvements in certain health outcomes associated with electronic patient portals [30-33], only a few were based in primary care [31,34]. Several systematic reviews that evaluated a variety of portals in different practice settings suggested that portals or similar digital health services may result in improved patient satisfaction, but they did not demonstrate a meaningful impact on health outcomes, cost, or use [35-40].

We found that providers spent less than 6 minutes per year on clerical work for each patient registered on the portal. This is a small amount of time per patient but is significant when considering the context of an entire primary care practice. We note that the time-stamping of messages was performed during the COVID-19 pandemic, while the number of messages sent by all providers was collected prior to 2020. We consider that even if the COVID-19 pandemic resulted in an increased number of messages, the provider time per message would not have changed significantly. Therefore, our estimate of portal-related clerical work reflects prepandemic time requirements, and these may have increased since 2020 due to increased patient interest in asynchronous virtual care. This would also be an area for further study. Portals that do not allow incoming messages or any secure messaging would reduce or eliminate this time requirement but might also limit patient engagement and other potential benefits of the portal. Since the clerical burden associated with electronic environments in health care has been associated with professional burnout, [22,26] it is important to consider the provider time requirement associated with patient portals. The time and cost associated with incorporating a patient portal are currently not specifically addressed in either fee-for-service or capitated Canadian primary care funding models.

There are other limitations to this study. We examined the long-term impact of an electronic patient portal in a single primary care practice, which may not be reflective of the impact in other primary care practices. However, portal adoption has not been widespread for long enough to allow study of the long-term impact of portals across multiple sites. Additionally, the impact of patient portals in other settings, such as hospitals, laboratories, or specialist practices, may be quite different. Further research is needed into electronic patient portals in different settings to determine their impact on various health outcomes.

### Conclusions

Electronic patient portals are increasingly being adopted by providers and sought after by patients. We found that portal registration was associated with a subsequent increase in the number of some traditional encounters and a small increase in clerical workload for providers. Portals have enormous potential to truly engage patients as partners in their own health care, but their impact on use of traditional health care services and clerical burden must also be considered when they are incorporated into primary care.

## Abbreviations

ITS: interrupted time-series
MD: medical doctor
NP: nurse practitioner
PA: physician assistant
TC: triage call
